# QuantiFERON^®^-TB Gold In-Tube for contact screening in BCG-vaccinated adults: A longitudinal cohort study

**DOI:** 10.1371/journal.pone.0183258

**Published:** 2017-08-30

**Authors:** Laura Muñoz, Lucia Gonzalez, Laura Soldevila, Jordi Dorca, Fernando Alcaide, Miguel Santin

**Affiliations:** 1 Infectious Diseases Department, Bellvitge University Hospital-IDIBELL, Barcelona, Spain; 2 Department of Clinical Sciences, University of Barcelona, Barcelona, Spain; 3 Respiratory Medicine, Bellvitge University Hospital-IDIBELL, Barcelona, Spain; 4 Microbiology Department, Bellvitge University Hospital-IDIBELL, Barcelona, Spain; 5 Department of Pathology and Experimental Therapeutics, University of Barcelona, Barcelona, Spain; Agencia de Salut Publica de Barcelona, SPAIN

## Abstract

**Objective:**

To assess the utility of QuantiFERON^®^-TB Gold In-tube (QFT-GIT) for targeting preventive therapy in BCG-vaccinated contacts of tuberculosis (TB), based on its high specificity and negative predictive value for development of TB.

**Methods:**

We compared two screening strategies for TB contact tracing in two consecutive periods: the tuberculin skin test (TST) period, when all contacts were screened with the TST alone; and the QFT-GIT period, when BCG-vaccinated contacts underwent TST and QFT-GIT. Diagnosis of TB infection among BCG-vaccinated contacts relied on TST ≥5 mm in the TST period, while in the QFT-GIT period either a positive QFT-GIT or a TST ≥15 mm was required.

**Measurements and main results:**

Six hundred and sixty-one contacts were compared. In the QFT-GIT period there was a reduction in diagnoses of TB infection (77.4% vs. 51.2%; *p* <0.01) and preventive therapy prescribed (62.1% vs. 48.2%; *p* = 0.02) among the 290 BCG-vaccinated contacts. After a median follow-up of 5 years, cumulative incidences of TB were 0.62 and 0.29 in the TST and QFT-GIT periods respectively (*p* = 0.59).

**Conclusions:**

In BCG-vaccinated TB contacts, the addition of QFT-GIT safely reduced TB diagnosis and treatment rates without increasing the risk of subsequent active TB.

## Introduction

Detection and treatment of recently infected people is an essential measure of tuberculosis (TB) control in low-prevalence countries [1]. Up to approximately ten years ago, the diagnosis of TB infection relied exclusively on the tuberculin skin test (TST). A positive TST response indicates infection with *Mycobacterium tuberculosis* indirectly, by measuring the delayed-type hypersensitivity response to the intradermal injection of a mixture of wall antigens, the so-called PPD (purified protein-derivate), which is shared by many mycobacteria species and the Bacillus Calmette–Guérin (BCG) strain [2]. The main limitations of the TST for targeting preventive therapy among the contacts of patients with pulmonary TB (TB contacts) are its low specificity and poor ability to identify those likely to develop active disease [3]. Thus, a high number of TB contacts need to be treated to prevent a case of TB in clinical practice.

By contrast to the TST, the interferon-γ release assays (IGRAs), the *in vitro* immunodiagnostic tests based on *M*. *tuberculosis* complex-specific antigens, have no cross-reactivity with the BCG-vaccine strains and most non-tuberculous mycobacteria [4–6]. After more than a decade, evidence indicates that, at best, the ability of these tests to predict the development of TB is only a little better than that of the TST [7, 8]. Nevertheless, IGRAs yield fewer positive results than TST, are more specific, and have shown a high negative predictive value for better selecting those immunocompetent individuals who will not develop TB; thus, their use for targeting TB contacts for preventive therapy, especially in BCG-vaccinated subjects, may still be preferable to TST and also more cost-effective in certain settings [9].

In 2007, the QuantiFERON^®^-TB Gold In-tube (QFT-GIT) test was implemented in our center. Shortly after, our TB Unit modified its internal protocol for contact tracing by adding the QFT-GIT to the ongoing TST-based strategy for screening and informing treatment decisions in BCG-vaccinated contacts of TB. Here, we report our experience with this practice. We hypothesized that using the QFT-GIT to target TB contacts would reduce the number of individuals diagnosed with, and treated for, TB infection compared with the previous TST-only strategy without an increased risk of subsequent active TB.

## Methods

### Design, setting, and study population

A retrospective comparative study of two screening strategies for TB contact tracing was performed at the TB Unit of a teaching hospital for adults in Barcelona (Spain) between January 2006 and December 2010. The Ethics Committee of Bellvitge University Hospital approved the study (PR269/11), and waived the need for consent. There was no specific survey questions or questionnaire, as data was gathered as part of the standard assistance protocol.

We included immunocompetent contacts older than 15 years who had no history of TB infection and whose index case had culture-proven non-MDR pulmonary TB. As part of routine clinical practice, medical histories, BCG-vaccination status (vaccine scar), treatment, adverse events and adherence to therapy had been gathered prospectively.

### Screening strategies and preventive therapy

We compared two consecutive 30-month periods: TST period (January 2006 to May 2008), and QFT-GIT period (June 2008 to December 2010). In both periods, active TB was ruled out through symptom-guided interview and chest X-ray. In the TST period there was no difference in contact management regarding BCG-vaccination status: the screening was performed with TST, and non-responders underwent a second test after the window period (8 weeks). In the absence of contraindications (p.e. pregnancy, liver disease), preventive therapy was prescribed if TST was ≥5 mm by 48–72 hours after administration. In the QFT-GIT period, two different strategies were used according to BCG-vaccination status. While non-BCG contacts were screened only with TST, as in the TST-period, BCG-vaccinated contacts were simultaneously screened using both the QFT-GIT assay and the TST. In this group preventive therapy indication was established by either a positive QFT-GIT assay or a TST result ≥15 mm. If the QFT-GIT assay was negative and the TST was <15 mm, a second QFT-GIT assay was performed 8 weeks later. Two trained nurses checked BCG-vaccine status through its characteristic scar and administered and read TST results. Treatment regimens included 6–9 months of treatment with isoniazid (INH) as the first-choice option, or 4 months with rifampicin (RMP) or 3 months with RMP plus INH as alternative regimens. While on treatment, contacts had regular appointments at the TB unit. There, both blood tests for monitoring liver function and adherence assessments were carried out. The latter included the Eidus-Hamilton test [10] for those taking INH and the simple checking of urine color for those on rifampicin.

### Follow-up

In 2015, the vital status and development of TB were checked among all contacts by retrospective review of the electronic medical records of both the Hospital and local primary care services, which were available online. If no data were available for the last 6 months or before the contact completed at least 5 years of follow-up, individuals were contacted by phone and briefly interviewed using a pre-designed questionnaire form. If the contact was not reachable, they were considered lost to follow-up. Contacts were censored at the time of active TB diagnosis, death, loss to follow-up, or after 5 years of follow-up, whichever came first.

### Data analysis

Incidence was given as the density of incidence (TB cases per person-time). Continuous variables were presented as medians (interquartile ranges) and compared with the Student *t* test or the Mann–Whitney *U* rank test, as appropriate. Differences in categorical variables were assessed with the χ^2^ test. All statistical analyses were two-tailed, and a *p*-value <0.05 was considered statistically significant. Analyses were performed with IBM^®^ SPSS^®^ Statistics for Macintosh, Version 22.0 (IBM Corp., Armonk, NY; released 2013) and the OpenEpi software (Open Source Epidemiologic Statistics for Public Health) [11].

## Results

**Fig 1:**
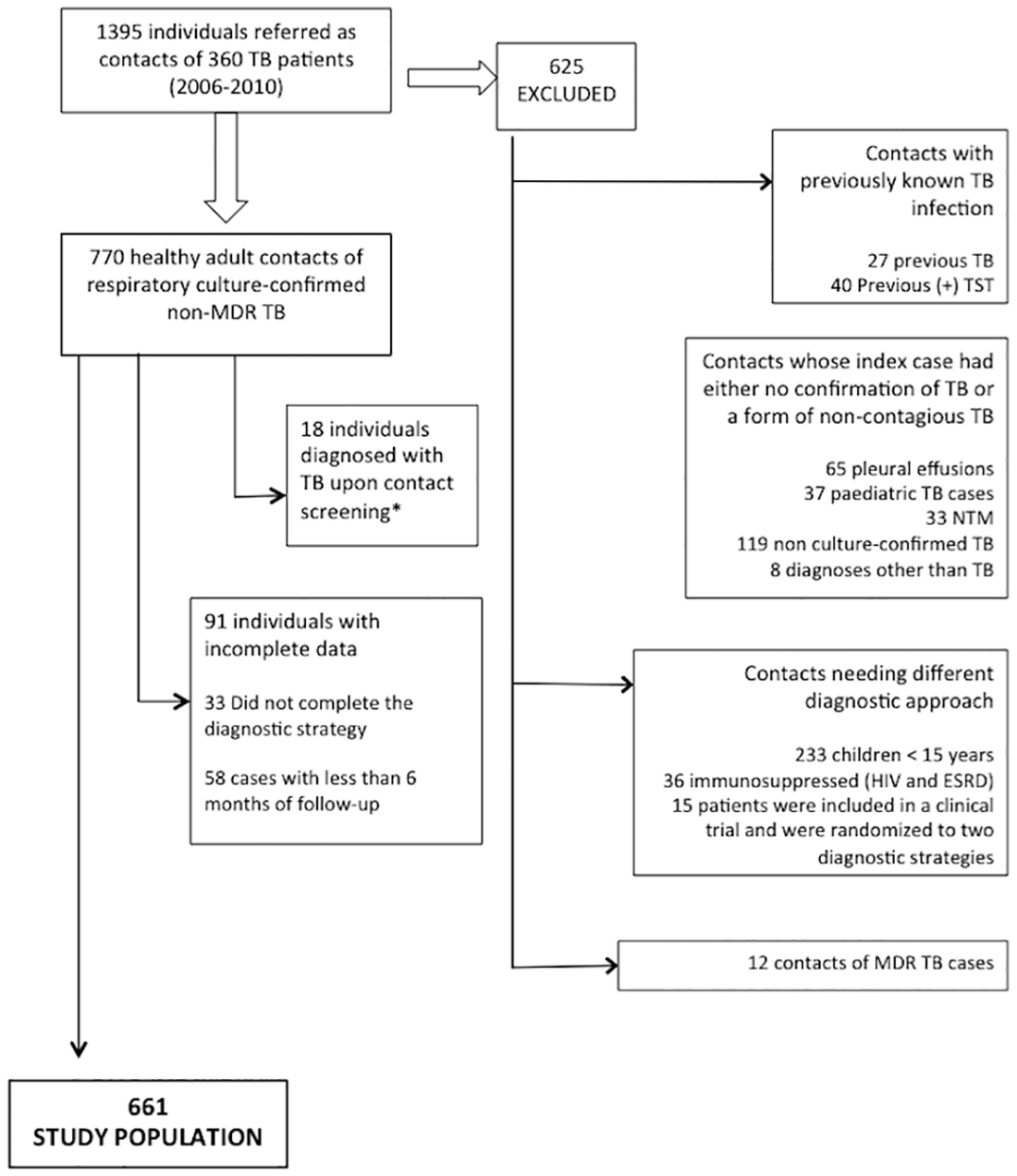
Chart of the contacts included. TB: tuberculosis; HIV: human immunodeficiency virus; MDR: multi-drug resistant; ESRD: end-stage renal disease; TST: tuberculin skin test; NTM: Non-tuberculous mycobacteria. ^a^12 and 6 patients in the first and second period, respectively.

During the study period, 1395 contacts of 360 index cases were evaluated, and 661 were included in the analysis (321 in the TST period and 340 in the QFT-GIT period). The selection of eligible contacts is summarized in Fig 1, and the baseline characteristics of the cohort by study period is shown in Table 1. The QFT-GIT period included a higher proportion of foreign-born individuals (*p* <0.01), close contacts (*p* <0.01), and BCG-vaccinated subjects (*p* = 0.01).

**Table 1:** Baseline characteristics and immunodiagnostic test results by study period.

	TST period (n = 321)	QFT-GIT period (n = 340)	*p*
Gender, man; n (%)	151 (47.0)	157 (46.2)	0.82
Age; median (IQR)	37 (25.5–48.5)	33.5 (21–46)	0.08
Foreign-born; n (%)	72 (22.4)	148 (43.5)	<0.01
-Latin America; n (% of foreign-born)	40 (55.6)	94 (63.5)	--
-North Africa	9 (12.5)	22 (14.9)	--
-Sub-Saharan Africa	1 (1.4)	15 (10.2)	--
-India/Pakistan	1 (1.4)	12 (8.1)	--
-South-East Asia	3 (4.2)	2 (1.4)	--
-Eastern Europe	18 (25)	3 (2)	--
Close contact^a^; n (%)	170 (53.0)	222 (65.3)	<0.01
Family ties with index case; n (%)	268 (83.5)	238 (70)	<0.01
Index case with positive smear; n (%)	229 (71.3)	228 (67.1)	0.24
BCG-vaccination; n (%)	124 (38.6)	166 (48.8)	0.01
1st TST-positive^b^; n (%)	194 (60.4)	168 (49.4)	<0.01
TB infection diagnosis; n (%)	223 (69.5)	184 (54.1)	<0.01
BCG-vaccinated	96/124 (77.4)	85/166 (51.2)	<0.01
Non-BCG-vaccinated	127/197 (64.5)	99/174 (56.9)	0.14
Preventive therapy prescribed; n (%)	186 (57.9)	171 (50.3)	0.05

IQR: interquartil range, BCG: Bacillus Calmette-Guérin, TST: tuberculin skin test.

^a^ Exposure to the index case was stratified as close (household or daily ≥6 hours of exposure), frequent (daily <6 hours of exposure), and occasional (no household or daily exposure, and <2 hours of exposure each time).

^b^ 1^st^ TST refers to the first TST result. If negative, a second test was carried out after the window period, established as 8 weeks.

### TB Infection diagnosis and preventive therapy

Regarding diagnosis, 407 of 661 contacts (61.6%) were diagnosed with TB infection according to the definition in each period (69.5% in the TST period and 54.1% in the QFT-GIT period; *p* <0.01). Of the 290 BCG-vaccinated contacts in both periods, 181 (64.5%) were diagnosed with TB infection (77.4% in the TST period and 51.2% in the QFT-GIT period; *p* <0.01). Table 2 shows the results of both tests for TB infection diagnosis in the 166 BCG-vaccinated patients in the QFT-GIT period. As for the 371 non-BCG contacts, 226 (60.9%) were diagnosed with TB infection, with no significant differences between periods (64.5% in the TST period and 56.9% in the QFT-GIT period; *p* = 0.14).

**Table 2:** TST and QFT-GIT results of BCG-vaccinated patients in the second period.

		QFT-GIT		
		Positive	Negative	
TST	Positive	55(40 (73%) patients with TST ≥15 mm)	44(21 (48%) patients with TST ≥15 mm)	99
	Negative	9	58	67
		64	102	166

TST: tuberculin skin test; QFT-GIT: QuantiFERON^®^-TB Gold In-Tube.

**Fig 2:**
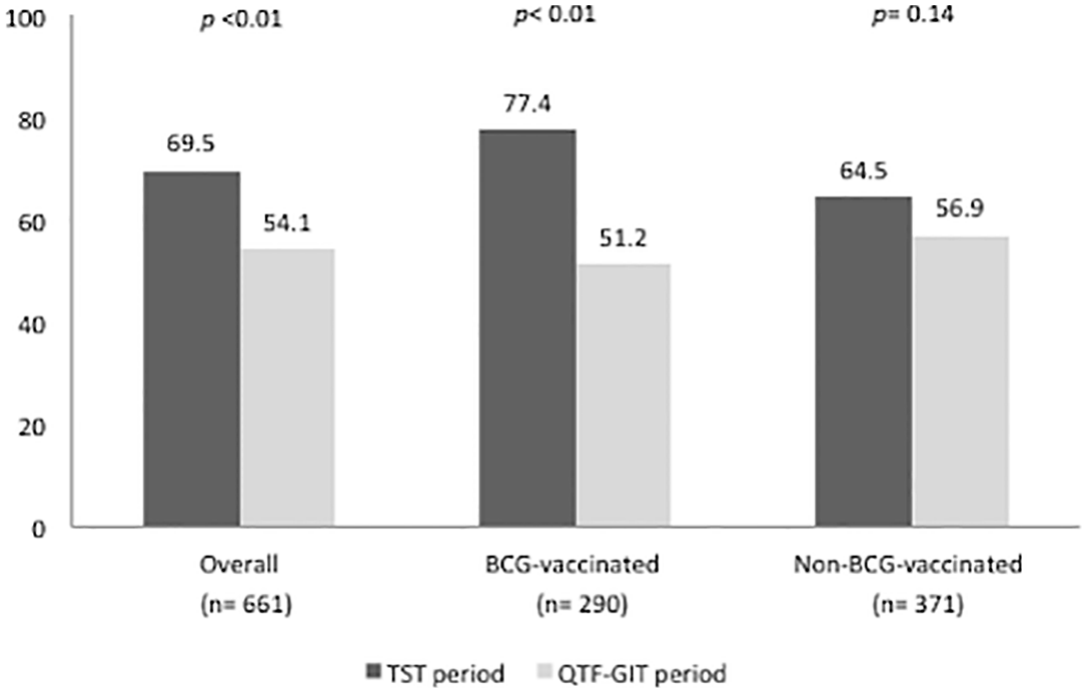
Proportion of TB infection diagnosis by study period and BCG-vaccination status.

While there was a higher proportion of diagnosis of TB infection among BCG-vaccinated contacts than among non-BCG-vaccinated contacts in the TST-period (77.4% and 64.5% respectively; *p* = 0.01), there was no such difference in the QFT-GIT period, when TB infection was diagnosed in 51.2% and 56.9% of BCG-vaccinated and non-BCG-vaccinated contacts respectively; *p* = 0.29) (Fig 2).

As regards treatment, 357 courses of preventive treatment were prescribed. There were 50 contacts diagnosed with TB infection that were not recommended treatment: 37 (16.6%) and 13 (7%) in the TST and QFT-GIT-periods respectively. Eight contacts refused treatment. The most common regimen was 6-month INH (n = 275), followed by INH for 9 months (n = 47), rifampicin for 4 months (n = 19), and combination therapy with INH-RMP for 3 months (n = 8). Among the 330 INH-based regimens, 19 individuals (5.8%) developed toxicity and required drug withdrawal; 14 of them (73.7%) completed treatment with RMP. Overall, 290 (81.2%) contacts completed a whole course of treatment (77.9% and 87.1% in the TST and QFT-GIT periods, respectively, p = 0.02). The higher proportion of lost to follow-up as well as rejection or early withdrawal of preventive treatment in the first period was associated to non-family ties with the index case (OR 2.63 [CI95% 1.43–4.84]).

### Development of active tuberculosis

The outcomes of the 661 contacts are shown in Table 3. Information was retrievable from the electronic medical records for 616 (93.2%) individuals, and another 45 (6.8%) were contacted by phone.

**Table 3:** Final disposition and incidence of active TB by study period.

	TST period (N = 321)	QFT-GIT period (N = 340)	*p*
Died^a^	11	13	0.79
Lost to follow-up before 5 years	14	54	<0.01
Median follow-up, years (IQR)	3.5 (2.0–4.2)	3.6 (2.7–4.4)	0.5
Median (IQR) follow-up, years (5 years maximum) ^b^	5 (N.A)	5 (4.9–5.0)	
Patient-years	1581.84	1595.15	
Incident TB cases	2	1	0.96
Cumulative incidence, %	0.62	0.29	0.59
Density of incidence,(TB cases x 1 000 p-years (95%CI))	1.26 (0.21–4.18)	0.63 (0.03–3.09)	0.62

TB: tuberculosis. TST: tuberculin skin test; QFT-GIT: QuantiFERON^®^-TB Gold In-Tube.

N.A: non-applicable.

^a^ Died of non-TB related causes.

^b^5 years maximum.

Over the median follow-up period of 5 years, three contacts developed active TB: two screened in the TST period and one screened in the QFT-GIT period. Table 4 shows their main features.

**Table 4:** Descriptive features of the TB cases diagnosed during the follow-up period.

	TST period		QFT-GIT period
Time from the first TB infection screening (years)	4.5	4.2	3.3
Epidemiologic data	Man, 51. Spanish	Woman, 22. Bolivian	Woman, 26. Spanish
BCG-vaccination	Yes	Yes	No
Relationship with the index case	Occasional relationship	Close contact	Close contact
TB infection screening	No TB infection: Negative TST (repeated after the window period) No treatment.	TB infection: Positive TST (16 mm). TB preventive treatment.	TB infection: Positive TST (13 mm): switched from negative after the window period). TB preventive treatment.
Risk factor for developing TB	No risk factors	Abandoned preventive treatment in the first month (pregnancy)	Lack of adherence to treatment (6-month isoniazid)
Form of TB	Pulmonary (upper lobes)	Pulmonary (upper lobes)	Pulmonary (upper lobes)

BCG: Bacillus Calmétte-Guerin; IC: Index case; TST: tuberculin skin test; TB: tuberculosis.

## Discussion

The results of this observational study support our hypothesis that the use of QFT-GIT for targeting BCG-vaccinated TB contacts for preventive therapy is as effective as a TST-based strategy for preventing subsequent development of TB, while allowing a substantial reduction of treatment prescriptions.

In 2008, we found that we were treating a huge proportion of BCG-vaccinated individuals (three out of every four contacts), and therefore decided to update the screening strategy. In June of that year we introduced the QFT-GIT assay as part of our routine assessment of BCG-vaccinated TB contacts, in line with current knowledge at that time. Despite the conservative approach of treating close contacts with TST ≥15 mm and negative QFT result, we attained a significant reduction of 26% in TB diagnoses among BCG-vaccinated contacts, without increasing the risk of active TB. This outcome is consistent with the findings of longitudinal studies in other countries with low and intermediate incidences of TB and high vaccination rates [12–14]. Although the effect of BCG vaccination on the TST’s specificity should not last over 10 years if BCG was received in infancy, as it used to until 1978 in Spain, and BCG would unlikely have a major influence in TST results in adults [15], obvious differences have been reported to date in TST results and BCG vaccination status [16].

In a German study of close contacts of smear-positive TB patients (n = 954), of the 495 BCG-vaccinated contacts who were TST-positive, 83% were ≥5 mm and 31% were ≥10 mm, while only 17% had a positive QFT-GIT assay result [12]. After at least two years of follow-up, none of the 413 TST-positive/QFT-GIT-negative untreated contacts had developed active TB. In a French study of 687 TB contacts, of the 300 TST-positive contacts (≥10 mm), only 106 (35%) had positive QFT-GIT results [13]. Two contacts developed active TB after 3 years, one of whom had a discordant TST-positive/QFT-GIT-negative result (negative predictive value for the QFT-GIT assay of 99.8%). In another retrospective study from South Korea, which included 1826 high-school student contacts, of the 270 TST-positive contacts (≥10 mm), 203 (75%) had positive QuantiFERON-Gold (QFT-G) results, but none of the 67 TST-positive/QFT-G-negative untreated contacts progressed to active TB [14]. Conversely, a Dutch study, which included foreign-born close contacts of smear-positive TB patients with high BCG-vaccination rates (81%), showed that using the QFT-GIT assay for preventive therapy decision, resulted in three missed contacts who had positive TST results and subsequently progressed to active TB [17]. However, in that study, QFT-GIT was performed only once, and shortly after the diagnosis of the index case. Therefore, it is plausible that these cases could have been captured if retested a few weeks later.

Despite the reported differences, the results of the present and the three previous studies indicate that the QFT-GIT assay is safe for targeting preventive therapy to fewer contacts. As for cost-effectiveness in contact screening, the benefits of applying QFT-GIT as either a confirmatory test or in place of the TST is also currently unknown. Some health economic models have explored this issue. Given the similar sensitivities of QFT-GIT and TST for TB infection in immunocompetent individuals and the higher specificity of QFT-GIT, despite its higher testing costs, some models indicate that IGRA-based strategies might be the most cost-effective option when a high pre-test probability is expected (>59%) [18]. However, in cases where the estimated probability is lower, performing the QFT-GIT only in TST-positive contacts would probably be most cost-effective, as it would significantly reduce the number of QFT-GIT tests, and thus the overall testing costs. In our study, the BCG-vaccinated group in the second period increased from 38% to almost half the cohort of contacts; the saving of 26% of preventive therapies, blood tests and follow-up visits, as well as the avoidance of unnecessary risk, justify the change in the protocol.

The present study also provides two relevant findings related to the screening and treatment of TB infection among contacts. First, there was a non-negligible TB prevalence of 2.3% at the time of the first appointment in the TB Unit among the 770 individuals with a recent infection in our cohort (12 and 6 patients in each period, respectively); indeed, this confirms the importance of contact tracing for finding new cases and providing early treatment to avoid TB transmission [19]. Second, a remarkably high proportion of individuals (81.2%) completed a full course of preventive therapy without serious adverse events. In 5% of cases, foreseeable liver toxicity was detected early and reversed by prompt drug withdrawal. Our experience confirms that high completion rates are possible when well-trained staff deliver comprehensive health education about treatment and toxicity following systematic interviews and providing written information [20].

Although the single-center design might theoretically be considered a limitation, in fact it is one of the main strengths of the study because it guarantees the homogeneity of the series. Since contacts were prospectively evaluated under the same clinical program, our results were not biased by diverse diagnosis and treatment strategies. Indeed, it was the evaluating team who treated the index cases and their contacts, prescribed preventive therapy, systematically took active measures to promote and control adherence to treatment, and looked for the follow-up of the contacts. While there are larger cohorts, they usually come from regional databases or the fusion of several databases coming from regions with different TB prevalence, with different diagnostic and therapeutic approaches and no complete data on BCG-vaccination [21]. This paper provides well-documented evidence on the secure saving of unnecessary treatments of TB contacts after the implementation of an IGRA. Although differences in LTBI diagnosis could be attributed to a difference in the cut-off of the TST (5 mm in the first period and 15 mm in the second), QFT-GIT contributed in the decision-making by means of its high negative predictive value [7].

The other strength of the study is the long-term assessment for TB development. These five years of follow-up include the highest risk period for developing active TB after being infected, which has been classically established in two years [22]. Had we chosen a two-year period, we would have missed the three new cases of TB.

Despite its strengths, this study also has limitations that deserve further comment. First, the retrospective design: development of active TB was evaluated by passive monitoring of contacts, who were assessed by reviewing clinical charts and phone interviews. This may have resulted in some missed cases of active disease, although specific features of TB (lack of mention in clinical notes equals lack of active TB) and the fact that active cases would have been referred to our Centre do make this unlikely. Moreover, we did a cross-match with the contacts in our cohort and the detection-system of new TB cases in Catalonia from 2006 to 2015. Second, there were differences in the follow-up at 5 years, probably because of the high proportion of immigrants in the second period (45.9%), who went back to their countries as a consequence of the economic crisis in our country. However, the mean follow-up period was almost 4 years (3.99; SD 1.08), which means that most of the high-risk period of developing TB was passed before leaving Spain. Third, there were very few contacts that progressed to active in both periods; as a consequence, wide confidence intervals impaired a proper comparison of TB incidence between the two periods. Fourth, since we did not genotype the causative strain of the three “assumed” cases of incident secondary TB, we cannot exclude the possibility of reinfection by a different strain.

In conclusion, the results of this study add evidence on the benefit of implementing QFT-GIT to target BCG-vaccinated contacts for preventive therapy. This approach reduces exposure to unnecessary treatment without increasing the risk of subsequent active TB. Prospective cohort studies with health economic data are needed to determine whether this strategy is suitable and cost-effective for the management of non-BCG-vaccinated contacts, and other risk groups for active TB.
